# Dexamethasone increases aquaporin-2 protein expression in *ex vivo* inner medullary collecting duct suspensions

**DOI:** 10.3389/fphys.2015.00310

**Published:** 2015-11-03

**Authors:** Minguang Chen, Hui Cai, Janet D. Klein, Oskar Laur, Guangping Chen

**Affiliations:** ^1^Division of Nephrology, The Second Affiliated Hospital and Yuying Children's Hospital of Wenzhou Medical UniversityWenzhou, China; ^2^Department of Physiology, Emory University School of MedicineAtlanta, GA, USA; ^3^Renal Division, Department of Medicine, Emory University School of MedicineAtlanta, GA, USA; ^4^Department of Microbiology and Immunology, Emory University School of MedicineAtlanta, GA, USA

**Keywords:** steroid, collecting duct, urine concentration, vasopressin, water homeostasis

## Abstract

Aquaporin-2 (AQP2) is the vasopressin-regulated water channel that controls renal water reabsorption and plays an important role in the maintenance of body water homeostasis. Excessive glucocorticoid as often seen in Cushing's syndrome causes water retention. However, whether and how glucocorticoid regulates AQP2 remains unclear. In this study, we examined the direct effect of dexamethasone on AQP2 protein expression and activity. Dexamethasone increased AQP2 protein abundance in rat inner medullary collecting duct (IMCD) suspensions. This was confirmed in HEK293 cells transfected with AQP2 cDNA. Cell surface protein biotinylation showed an increase of dexamethasone-induced cell membrane AQP2 expression and this effect was blocked by glucocorticoid receptor antagonist RU486. Functionally, dexamethasone treatment of oocytes injected with an AQP2 cRNA increased water transport activity as judged by cell rupture time in a hypo-osmotic solution (66 ± 13 s in dexamethasone vs. 101 ± 11 s in control, *n* = 15). We further found that dexamethasone treatment reduced AQP2 protein degradation, which could result in an increase of AQP2 protein. Interestingly, dexamethasone promoted cell membrane AQP2 moving to less buoyant lipid raft submicrodomains. Taken together, our data demonstrate that dexamethasone promotes AQP2 protein expression and increases water permeability mainly via inhibition of AQP2 protein degradation. The increase in AQP2 activity promotes water reabsorption, which may contribute to glucocorticoid-induced water retention and hypertension.

## Introduction

Dexamethasone is a synthetic glucocorticoid that has been used clinically since the early 1960's to treat a variety of conditions. A major adverse effect of dexamethasone treatment is hypertension (Kalimi, 1989; Ferrari, 2003; Mangos et al., 2003; Li et al., 2008). However, the underlying mechanism of dexamethasone-induced hypertension remains largely unknown. In animal studies, administration of dexamethasone in the drinking water induces a rapid and sustained elevation of blood pressure in wild-type mice (Zhang et al., 2009; Goodwin et al., 2010). This effect is mediated via the glucocorticoid receptor and is independent of the mineralocorticoid receptor (Zhang et al., 2009; Goodwin et al., 2010). Interestingly, the dexamethasone-induced blood pressure increase is not accompanied by an increase in total peripheral resistance (Ong et al., 2009) and sodium retention (Ong et al., 2007).

The water channel aquaporin 2 (AQP2) expressed in the apical membrane of the principal cells of renal collecting duct, mediates water absorption, and plays a crucial role in maintaining water homeostasis in mammals (Moeller et al., 2010; Chen et al., 2012). The global AQP2 knockout mice died within the first few days of life (Rojek et al., 2006). The connecting tubule specific AQP2 knockout mice have defective renal water handling under basal conditions, with higher urine volumes and reduced urine osmolality (Kortenoeven et al., 2013). Dysfunction of AQP2 is linked to many water related diseases, such as central and nephrogenic diabetes insipidus, chronic heart failure, and nephrotic syndrome (Jaffuel et al., 2013). Arginine vasopressin (AVP, also named antidiuretic hormone, ADH) is the major hormone that regulates AQP2 activity *in vivo* (Saito et al., 2009; Fenton et al., 2013). Vasopressin binds to the V_2_ vasopressin receptor on the basolateral side of collecting duct principal cells and activates a cAMP-dependent signal transduction pathway. Acute regulation by vasopressin involves AQP2 phosphorylation and translocation from cytoplasmic vesicles to the apical plasma membrane in the principal cells of the collecting duct (Tamma et al., 2011; Radin et al., 2012), and the subsequent increase in the water permeability of the epithelium. Chronic vasopressin increases AQP2 protein abundance in the collecting duct. AQP2 abundance is increased about 4–10-fold after long-term increases in circulating vasopressin levels (Radin et al., 2012).

A number of studies have shown that glucocorticoid hormones modulate ion channels and transporter activities that could regulate water balance and blood pressure. Dexamethasone up-regulates ENaC (Sayegh et al., 1999) and CFTR (Caohuy et al., 2009; Prota et al., 2012) expression at both transcriptional and post-transcriptional levels. Dexamethasone increases the transcript level and the membrane protein abundance of the Na^+^/Ca^2+^ exchanger NCX3 (Heise et al., 2011). We and Schrier showed downregulation of the urea transporter UT-A1 by glucocorticoids (Klein et al., 1997; Naruse et al., 1997; Li et al., 2008). Previous study from animal experiments showed that dexamethasone significantly upregulated the expression of AQP2 in the inner medulla in adrenalectomized rats (Chen et al., 2005). However, whether and how dexamethasone can directly regulate AQP2 expression and water permeability in kidney inner medullary collecting duct (IMCD) is still unknown.

To avoid the extra-renal environmental factors that might influence the effects of dexamethasone on AQP2, in this study we examined the role of dexamethasone on AQP2 protein expression by using rat IMCD suspensions and HEK293 cells transfected with an AQP2 cDNA. The functional effect of dexamethasone on AQP2 was examined by using the oocyte expression system. We found that dexamethasone increases AQP2 expression in rat IMCD suspensions and in the cultured HEK293 cells and increases AQP2 activity in *Xenopus* laevis oocytes. This effect occurs mainly through inhibition of AQP2 protein degradation.

## Materials and methods

### Animal experiments

Male Sprague-Dawley rats (Charles River Laboratories, Wilmington, MA) weighing 200–300 g received free access to water and standard rat chow. The rats were euthanized by CO_2_ asphyxiation. Kidneys were removed and the inner medulla (IM) was dissected. IMCD suspensions were prepared by digestion with hyaluronidase and collagenase B (Sigma, St. Louis, MO) as described before (Chen et al., 2011) and incubated with DMEM supplemented with 10% fetal bovine serum (FCS), 25 mM HEPES in a 5% CO_2_ incubator. The freshly prepared suspensions were treated with dexamethasone (Sigma, D8893), vasopressin (Sigma, V9879), or forskolin (Sigma, F3917) for the indicated times and subjected to lipid raft isolation or protein analysis by Western blot. All animal protocols were approved by the Emory University Institutional Animal Care and Use Committee.

### Plasmid construction

The rat AQP2 cDNA gene coding the full-length protein was RT-PCR amplified from kidney IM mRNA. The primers were designed based on the AQP2 gene (access number: NM 012909) and a BamHI site was introduced in forward primer AQP2a: 5′-CGGGATCCGGAG CAGCATGTGGGAACTCAGATCC -3′ and a XbaI site was added in reverse primer AQP2b: GCTCTAGAGGGAGCTCAGGCCTTGCTGCCGCGAG. The PCR amplified product was purified, digested with BamHI/XbaI and subcloned into a mammalian expression vector pcDNA3 (pcDNA3-AQP2) or into an oocyte expression vector pGH19 (pGH19-AQP2). All of the constructs were verified by nucleotide sequence analysis.

### Cell culture, transient transfection, and treatment

HEK293 cells were routinely cultured in DMEM supplemented with 10% FCS at 37°C in 5% CO_2_. Plasmids were transfected by Lipofectamine 2000 (Invitrogen) according to the instruction manual. The cells were treated with 0.1 μM dexamethasone, 0.1 μM RU486 (or mifepristone), 0.1 μM vasopressin, or 10 μM forskolin for the indicated times. For the protein degradation studies, the cells were incubated with 100 μg/ml cycloheximide (Sigma, C4859) and chased for up to 12 h.

### Cell surface protein biotinylation

Cell surface biotinylation assays were performed as described before (Chen et al., 2006). Briefly, after treatment, the cells were incubated twice with a freshly prepared solution of 1.0 mg/ml EZ-Link sulfo-N-hydroxysuccinimide disulfide-biotin (Pierce, 21331) in borate buffer for 30 min at 4°C. The biotin reaction was quenched for 5 min with 0.1 mM lysine (Sigma, L5626). After washing with PBS, the cells were lysed in a modified radioimmuno-precipitation assay (RIPA) buffer (150 mM NaCl, 10 mM Tris.HCl, pH 7.5, 1 mM EDTA, 1% Triton X-100, 1% sodium deoxycholate, 0.1% SDS, and protease inhibitors). Equal amounts of cleared lysate protein (0.5–1 mg) were incubated with 25 μl of immobilized streptavidin-agarose beads (Pierce, 20349) overnight at 4°C with gentle shaking. The beads were washed four times with RIPA buffer. Biotin-labeled proteins were eluted in 35 μl of Laemmli sample buffer and analyzed for AQP2 expression by Western blotting.

### Cell lysate preparation and Western blot

After treatment, the IMCD suspensions or HEK293 cells were homogenized in a glass homogenizer in RIPA buffer. The supernatants were collected after a brief centrifugation at 10,000 rpm for 10 min at 4°C. The protein concentration was determined by the BCA Protein Assay (Thermo scientific 23223). For Western blot analysis, the proteins were separated by 10% or 4–15% SDS-PAGE and electrotransferred to polyvinylidene difluoride (PVDF) membranes (Bio-Rad). The membranes were routinely processed by blocking with 5% milk/PBST, incubation with primary antibody overnight and with horseradish peroxidase-conjugated secondary antibody for 1 h. Proteins were detected using an Enhanced Chemiluminescence (ECL) Kit (Amersham). The following antibodies were used: AQP2 (Kim et al., 2003); UT-A1 (Chen et al., 2011); GAPDH (Santa Cruz, sc-25778); secondary horseradish peroxidase-conjugated goat anti-rabbit IgG (Amersham, NA934). The Western blot signals were quantified using the NIH ImageJ program. The intensity of AQP2 was normalized to GAPDH and expressed as a percentage compared with the value in controls (100%).

### Oocyte isolation, cRNA preparation and microinjection, biotinylation and water permeability experiments

Xenopus laevis oocytes were prepared and maintained in OR3 medium as described previously (Chen et al., 2011). Capped AQP2 cRNAs were transcribed from linearized pGH19-AQP2 with T7 polymerase using the mMESSAGE mMACHINE T7 Ultra Kit (Ambion). Two ng of AQP2 cRNAs were injected into oocytes. After 3 days, good and healthy oocytes were selected for protein expression and functional measurements. Some cells were treated with 0.1 μM dexamethasone for 2 days. UT-A1 cRNAs (Chen et al., 2011) were also injected and used as a control. Oocyte biotinylation was performed as before (Feng et al., 2009). Oocyte water permeability was measured by placing oocytes in a low osmotic solution (one volume of ND96 mixed with one volume of water), and the time to cell rupture in the hypo-osmolarity solution was counted by visual inspection using a microscope.

### Isolation of lipid rafts from IMCD

IMCD lipid raft fractionations were performed with a 5–40% sucrose discontinuous gradient as reported previously (Chen et al., 2011). Briefly, after dexamethasone treatment, rat IMCD suspensions were homogenized in 0.5% Brij 96V (Sigma)/TNEV buffer (10 mM Tris-HCl pH 7.5, 150 mM NaCl, 5 mM EDTA, 2 mM Na vanadate, and protease inhibitor cocktail) on ice for 30 min. The supernatants were collected and mixed with 80% sucrose by 1:1 volume. The sucrose gradient was built by layering on the top with 35 and 5% sucrose and then centrifuged in a SW 50.1 rotor (Beckman Coulter) at 34,000 rpm (~110,000 g) for 16 h at 4°C. Equal sizes of fractions (~400 μl) were collected from the top to bottom and analyzed by Western blot.

### Statistical analysis

All values were expressed as means ± SD. Statistical analysis of the data was performed by One-Way ANOVA followed by Tukey's HSD test. Differences were considered as significant at *P* < 0.05.

## Results

### Dexamethasone increases AQP2 protein abundance in rat IMCD

**Figure 1:**
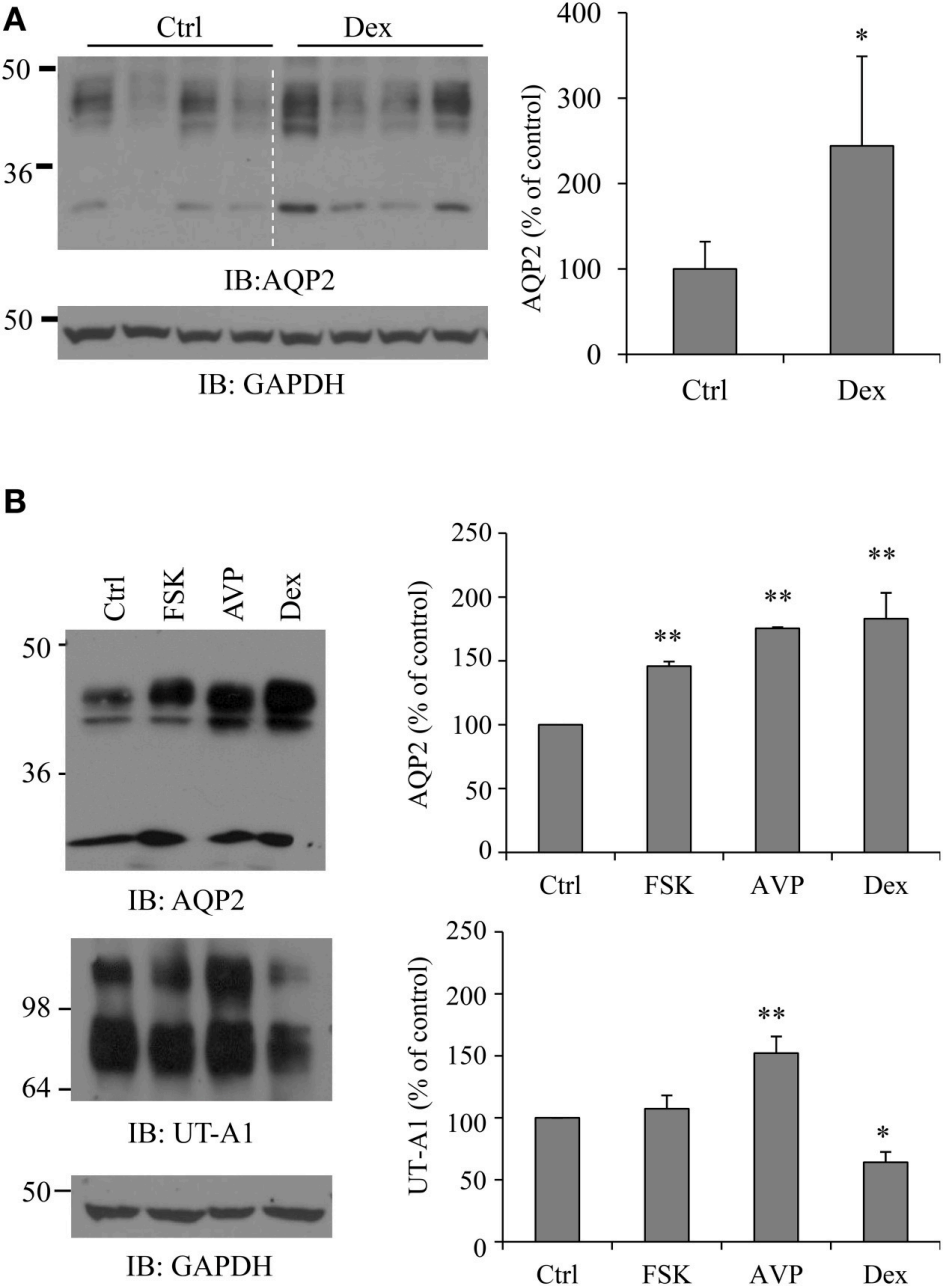
**Dexamethasone (Dex) treatment increases AQP2 protein abundance in rat IMCD**. **(A)** Rat IMCD suspensions were exposed to 0.1 μM Dex for 6 h and lysed in RIPA buffer. Equal amounts of supernatant were immunoblotted with antibodies to AQP2 and GAPDH. **(B)** Rat IMCD suspensions were exposed to 0.1 μM AVP, 10 μM forskolin (FSK) or 0.1 μM Dex. Equal amounts of lysates were immunoblotted with antibodies to AQP2, UT-A1, and GAPDH. Western blot signals were quantified by NIH ImageJ (compared to control, ^*^P < 0.05, ^**^P < 0.01, *n* = 3).

To investigate the direct effect of dexamethasone on AQP2 protein expression in kidney IM, freshly prepared IMCD suspensions were exposed to 0.1 μM dexamethasone for 6 h. IMCD pellets were then lysed in RIPA buffer. Equal amounts of cell lysate were immunoblotted with antibodies to AQP2 and GAPDH. Western blot signals were quantified by NIH ImageJ. Kidney AQP2 shows a narrow 29 kDa band and 35–50 kDa diffuse bands. Densitometry quantification included both forms. Figure 1A shows that dexamethasone treatment increases total AQP2 protein abundance (*p* < 0.05, *n* = 4). Since vasopressin upregulates AQP2 (Hasler et al., 2002; Nedvetsky et al., 2010), we also treated IMCD suspensions with vasopressin and adenylyl cyclase stimulator forskolin. Figure 1B shows that dexamethasone, as well as vasopressin and forskolin, upregulated AQP2 protein expressions. Urea transporter UT-A1 is another important transporter expressed in kidney IMCD and is regulated by vasopressin. Early studies by us and Schrier et al. showed that glucocorticoid (dexamethasone) treatment downregulates UT-A1 protein expression (Klein et al., 1997; Naruse et al., 1997; Li et al., 2008). The same samples were examined for UT-A1 protein expression. Unlike upregulating AQP2, dexamethasone treatment caused a decrease of UT-A1 protein abundance. Native UT-A1 from kidney presents two glycosylation forms of 97- and 117-kDa bands. Both bands were included in the densitometry analysis.

### Dexamethasone upregulates AQP2 expression in the transfected HEK 293 cells

**Figure 2:**
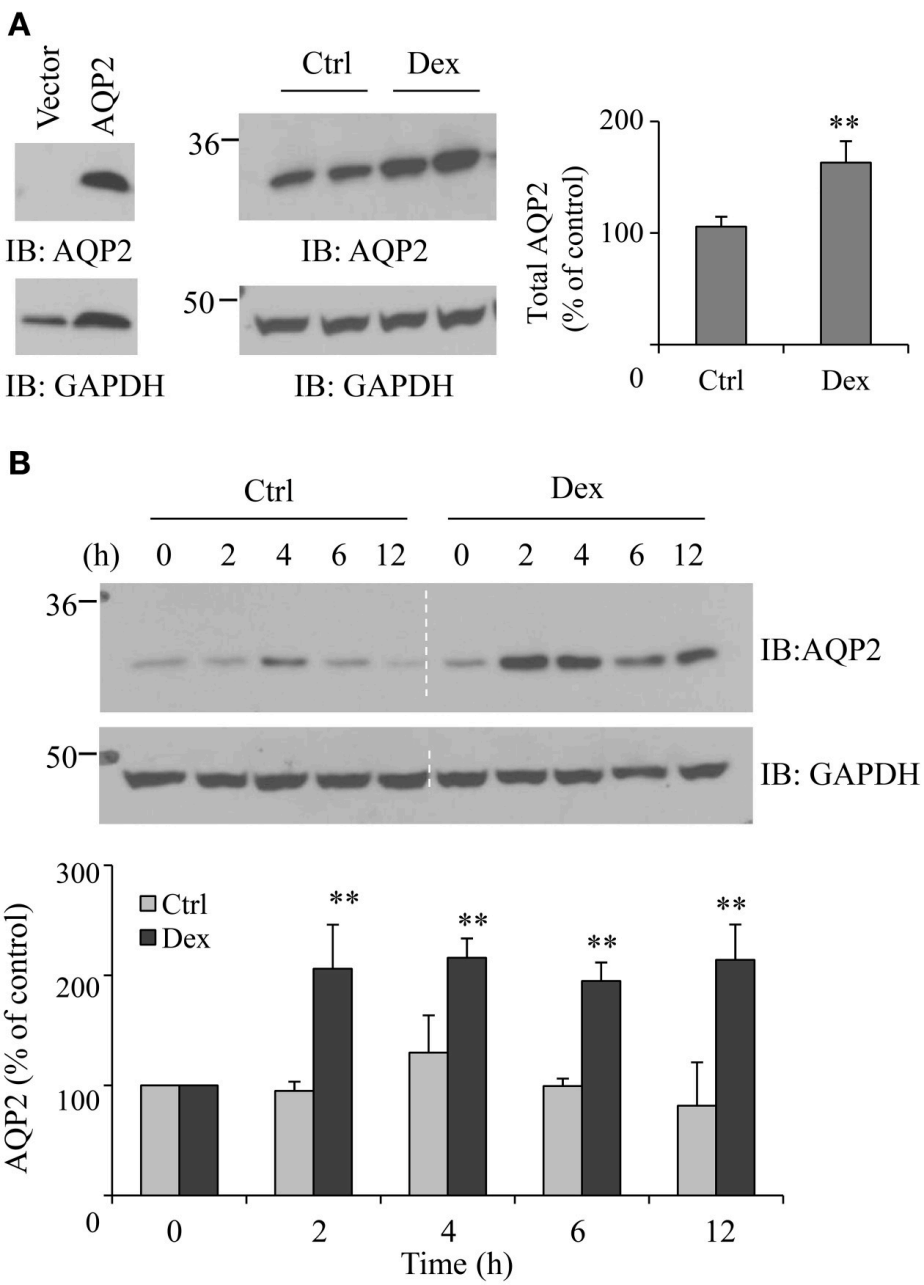
**Dexamethasone increases AQP2 expression in the transfected HEK 293 cell**. **(A)** HEK293 cells were grown in 6-well plates and transfected with pcDNA3-AQP2 1.0 μg for 48 h. After incubation with 0.1 μM Dex for 6 h, cells were collected and solubilized in RIPA buffer. Equal amounts of proteins in the supernatant were analyzed by immunoblotting with AQP2 and GAPDH antibodies (*n* = 4). **(B)** Time course experiment. HEK293 cells were transfected with pcDNA3-AQP2 1.0 μg for 48 h then treated by 0.1 μM Dex for the indicated time. AQP2 protein expression was analyzed by immunoblotting with AQP2 antibody. Western blot signals were quantified by NIH ImageJ (compared to control, ^**^P < 0.01, *n* = 4).

To further evaluate the influence of dexamethasone on AQP2 protein expression, we used HEK293 cells transiently transfected with pcDNA3-AQP2 cDNA. Forty-eight h after transfection, cells were incubated with 0.1 μM dexamethasone for 6 h. As observed in rat IMCD suspensions, dexamethasone treatment increased AQP2 protein abundance by ~63% (*p* < 0.01, *n* = 3) (Figure 2A). The time course studies revealed that the effect of dexamethasone on AQP2 protein expression started as early as 2 h after treatment (Figure 2B).

### Dexamethasone increases AQP2 cell membrane expression

**Figure 3:**
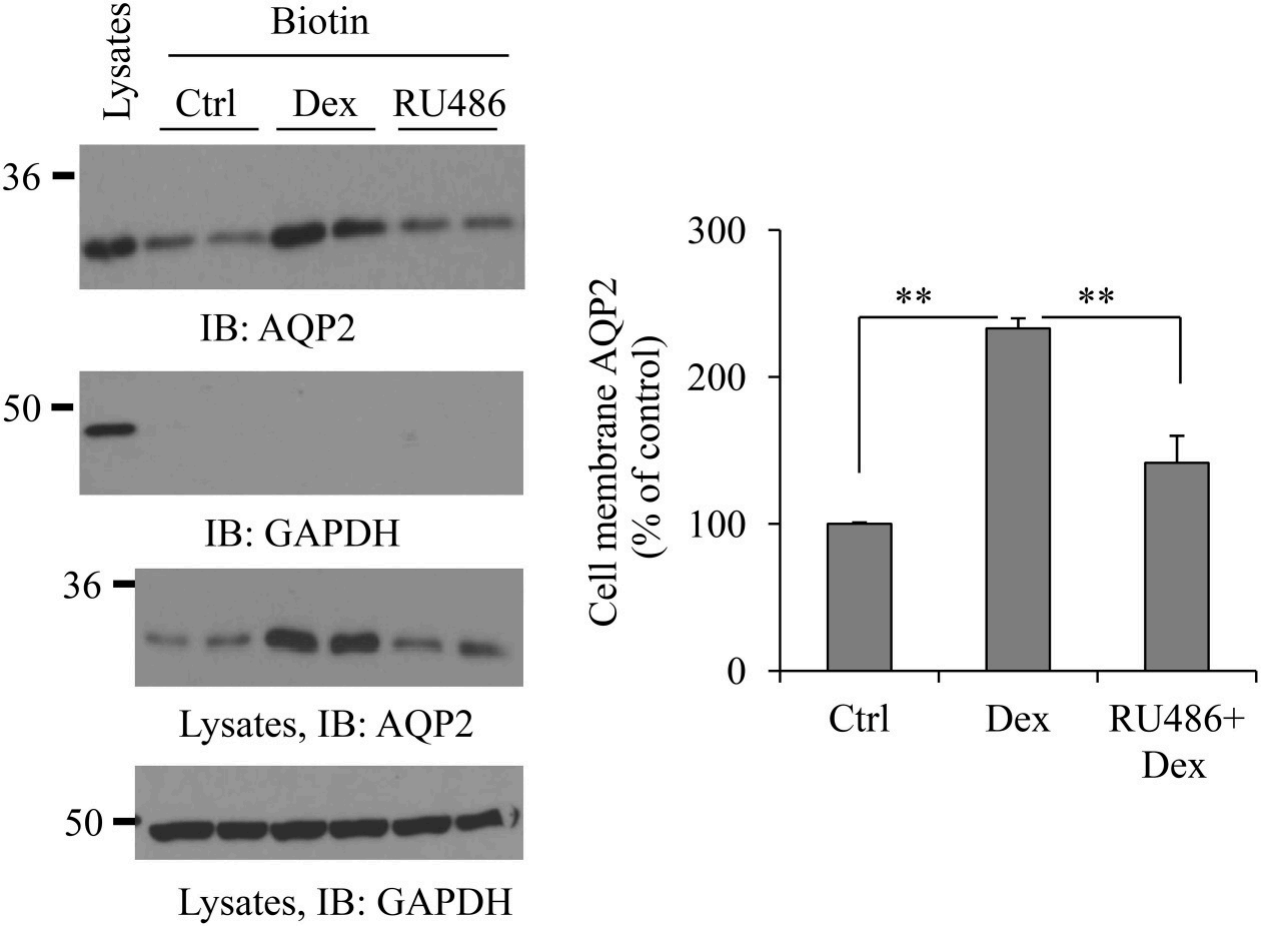
**Dexamethasone increases AQP2 cell membrane expression**. HEK293 cells were grown in 6-well plates and transfected with pcDNA3-AQP2 1.0 μg for 48 h. After incubation with Dex or 0.1 μM RU486 pre-treatment 30 min + Dex for 6 h, cells were processed for cell membrane protein biotinylation and lysed in RIPA buffer. The biotinylated proteins were collected by streptavidin beads. Cell membrane AQP2 was analyzed by immunoblotting with AQP2 antibody. Western blot signals were quantified by NIH ImageJ (compared to control, ^**^P < 0.01, *n* = 4).

To examine whether dexamethasone treatment could increase AQP2 cell membrane abundance, cell surface protein biotinylation was carried out. Figure 3 shows an increase of dexamethasone-induced cell membrane AQP2 expression. To investigate whether this effect is mediated through the glucocorticoid receptor, some cells were pre-treated with 0.1 μM glucocorticoid receptor antagonist RU486 (Ogata et al., 2001) for 30 min, then treated with dexamethasone. RU486 blocked total and cell membrane AQP2 abundance induced by dexamethasone.

### Dexamethasone increases AQP2 activity in oocytes

**Figure 4:**
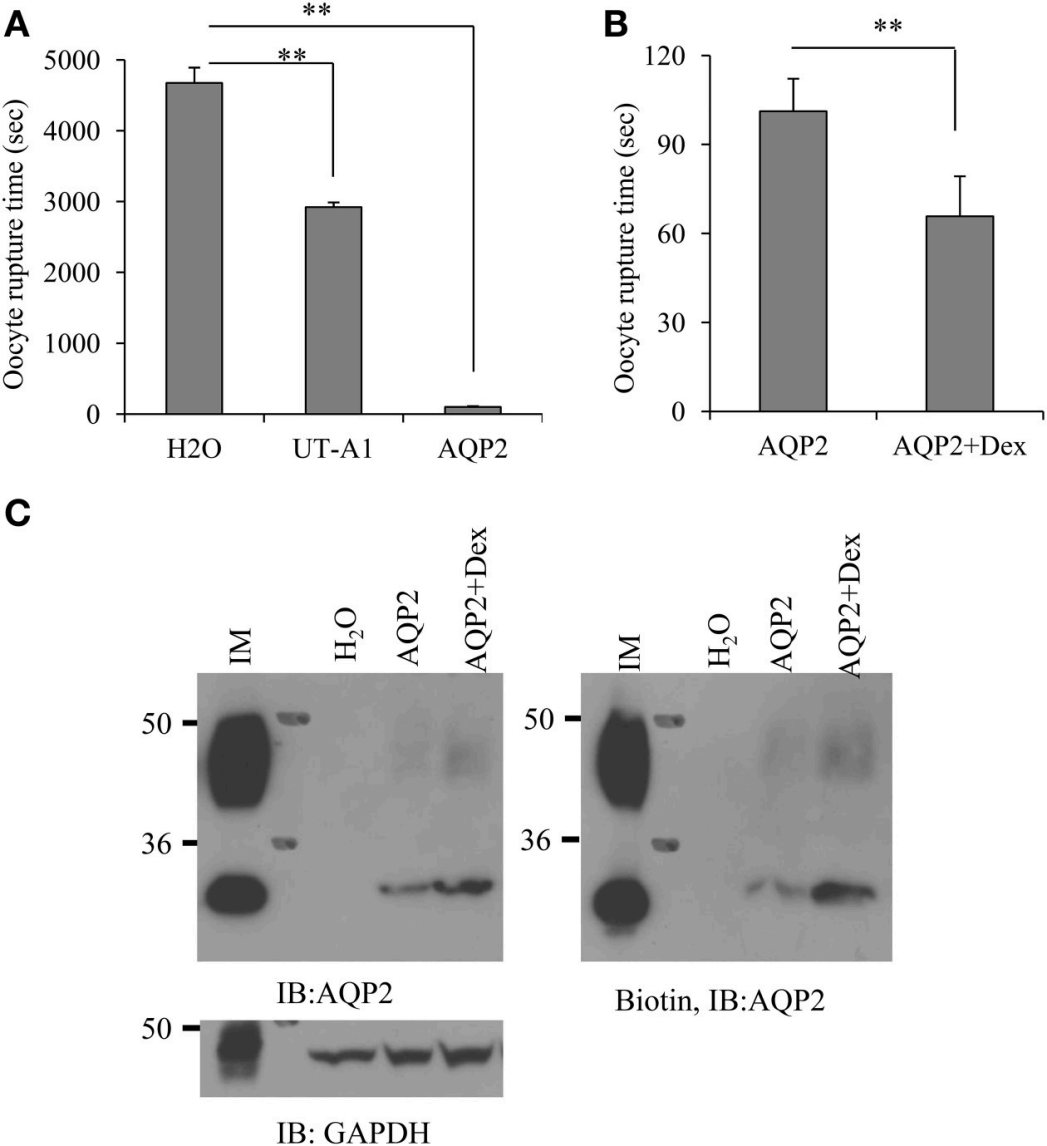
**Dexamethasone treatment increases AQP2 protein expression and water permeability in oocytes**. Oocytes were injected with cRNA encoding AQP2 (2 ng/23 nl/cell) or UT-A1 (2 ng/23 nl/cell). Control cells were injected with the same amount of water. Some AQP2 injected oocytes were incubated with 0.1 μM dexamethasone for 48 h. **(A,B)** The water permeability was measured by counting cell rupture time in a hypo-osmotic solution (^**^P < 0.01, *n* = 15). **(C)** AQP2 protein expression. Oocytes (*n* = 15) were processed for cell surface biotinylation. Total and biotinylated AQP2 were examined by western blot with AQP2 antibody. Kidney IM tissue was used as a positive control.

To assess whether the increased AQP2 protein expression following treatment by dexamethasone correlates with a functional change in water transport, AQP2 was expressed in oocytes. AQP2 water transport activity was measured by counting cell rupture time when the cells were switched from 200 mOsm to a hypo-osmotic solution of 100 mOsm. The average cell rupture time for the water-injected control oocytes is about 4675.2 ± 216.7 s (*n* = 15). The cell rupture time for the cells injected by AQP2 is 101.2 ± 11 s (*n* = 15). We also injected UT-A1 cRNA as a control. Although the average rupture time for UT-A1 injected oocytes was 2920.8 ± 67.4 s (*n* = 15), much longer than that of AQP2 injected oocytes, it is significantly shorter than that in water-injected oocytes (*p* < 0.01), indicating that UT-A1 somehow weakly moves water (Figure 4A). The water permeability experiment in Figure 4B showed that the cell rupture time in the dexamethasone-treated AQP2 cells was 65.8 ± 13.5 s shorter than that in the untreated AQP2 cells 101.2 ± 11 s (*P* < 0.01, *n* = 15), indicating that dexamethasone treatment increases AQP2 activity. AQP2 protein expression was examined by biotinylation and western blot. Compared to the untreated AQP2 cRNA injected cells, the total and cell membrane AQP2 protein were all increased by dexamethasone (Figure 4C).

### Dexamethasone reduces AQP2 degradation

**Figure 5:**
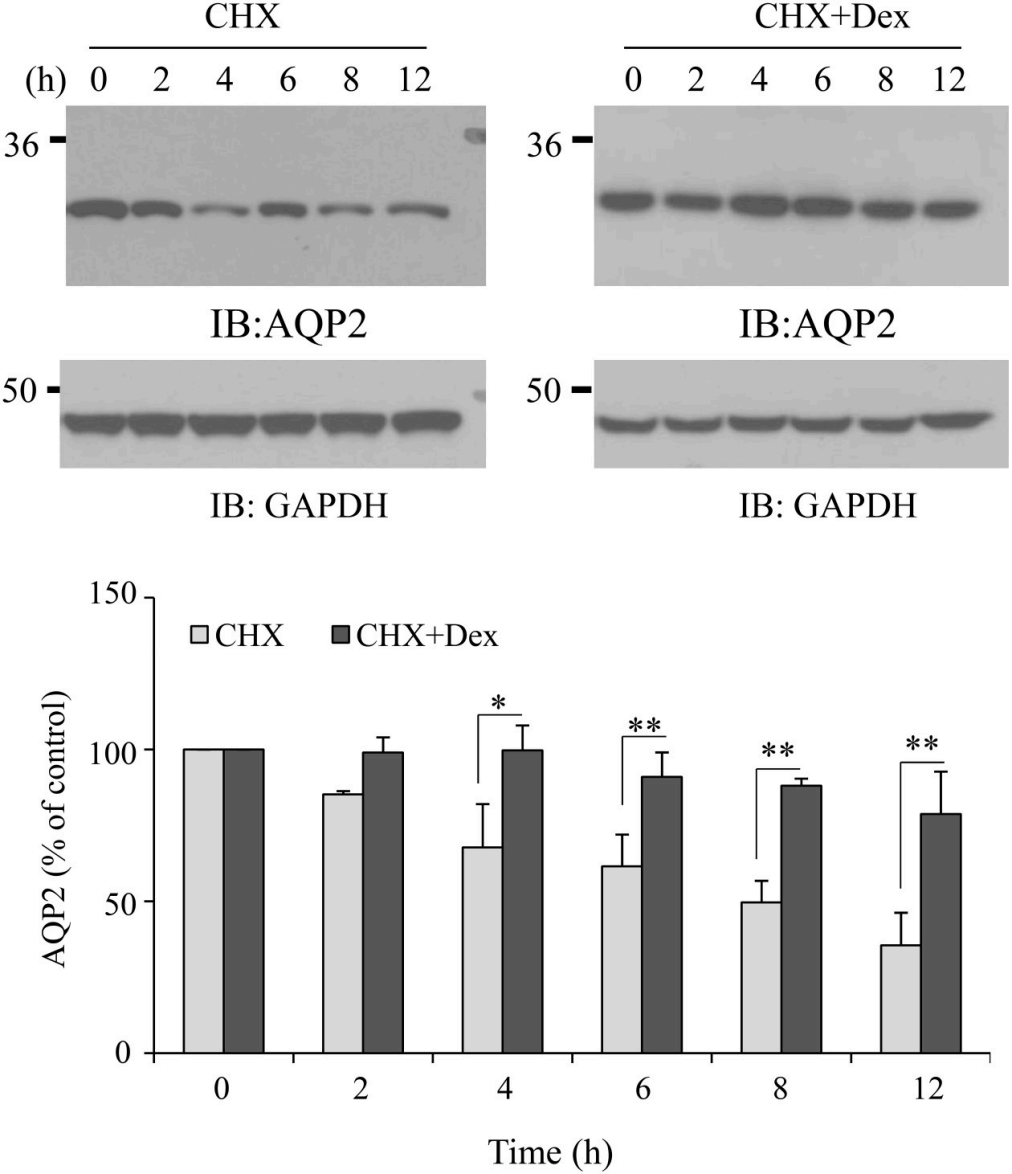
**Effect of dexamethasone on AQP2 stability**. HEK293 cells were transfected with pcDNA3-AQP2. After 48 h, the cells were pre-treated with CHX (100 μg/ml) for 30 min, then treated without or with dexamethasone (0.1 μM) for the indicated time. Total AQP2 protein was analyzed by western blot. Protein signals were quantified by NIH ImageJ (compared to CHX, ^*^P < 0.05, ^**^P < 0.01, *n* = 3).

To address the possible mechanism by which dexamethasone upregulates AQP2, we investigated AQP2 degradation mediated by dexamethasone. AQP2 HEK293 cells were pre-treated with cycloheximide (CHX) to inhibit new protein synthesis. AQP2 protein degradation was assessed in the presence or absence of dexamethasone. Comparing with non-dexamethasone-treated control cells, AQP2 degradation was significantly reduced with dexamethasone treatment (Figure 5). This suggests that dexamethasone increases AQP2 level, at least partially, by decreasing AQP2 degradation.

### Dexamethasone changes AQP2 distribution in lipid rafts

**Figure 6:**
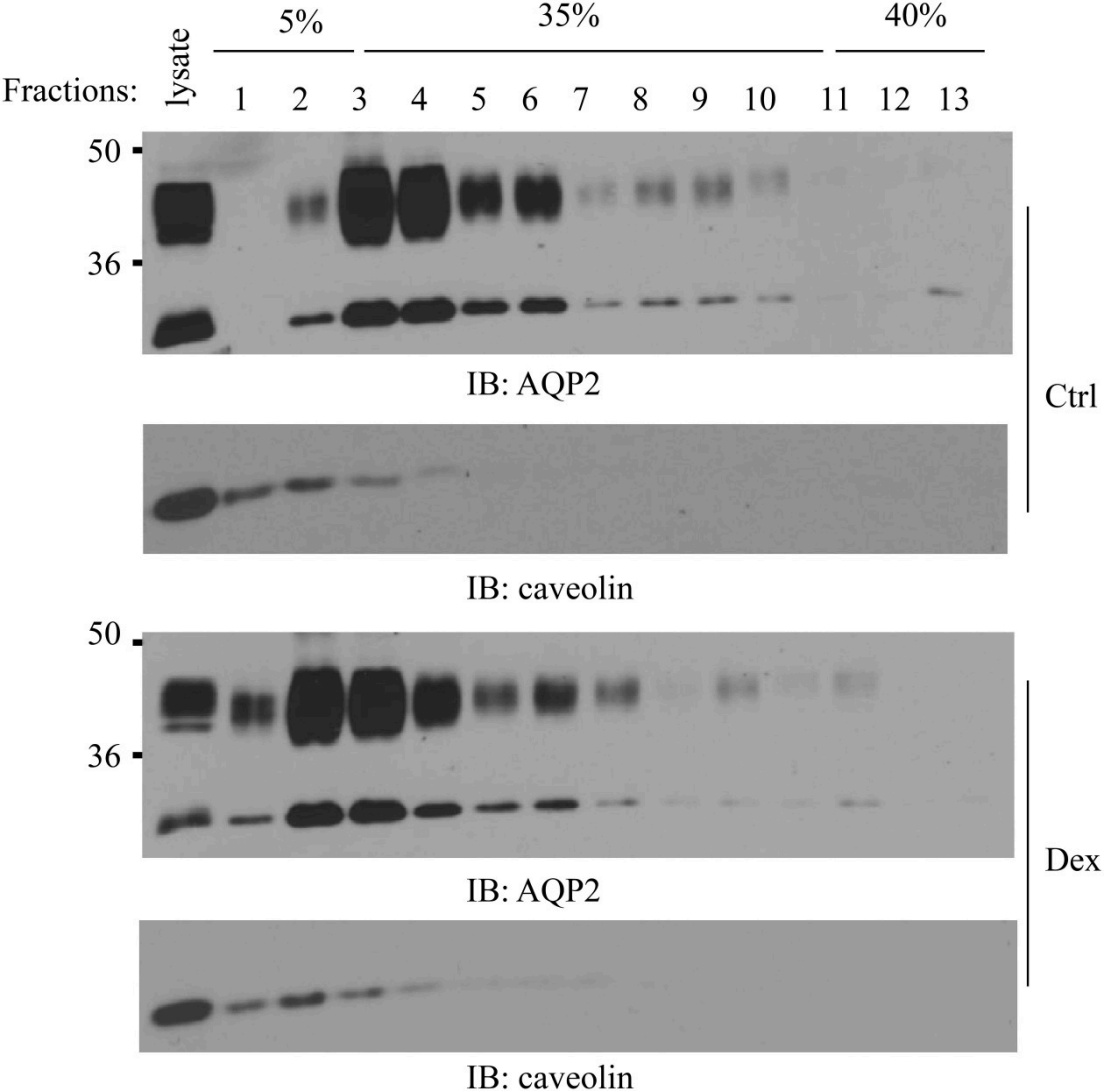
**Effect of dexamethasone on AQP2 lipid raft distribution**. Kidney IMCD suspensions were treated with/without 0.1 μM dexamethasone for 6 h then processed for lipid raft isolation by a 5–40% sucrose gradient ultracentrifugation. Equal sizes of fractions (~400 μl) were collected from the top to bottom and analyzed by Western blot with AQP2 and caveolin-1 antibodies (*n* = 3).

We previously reported that AQP2 is localized in cell membrane lipid raft submicrodomains (Feng et al., 2009). Many membrane proteins' apical sorting is determined by their association with lipid rafts (Simons and Ikonen, 1997; Brown and London, 1998). We treated IMCD suspensions with dexamethasone for 6 h and then examined whether dexamethasone could alter AQP2 distribution in lipid rafts. Caveolin-1 was used as a positive control for lipid raft fractions (Feng et al., 2009). Interestingly, dexamethasone treatment increased AQP2 movement into less buoyant lipid raft fractions 1~2 (Figure 6).

## Discussion

Glucocorticoid hormones affect body water homeostasis (Ferrari, 2003). Many ion channels and transporter, such as ENaC (Sayegh et al., 1999), CFTR (Caohuy et al., 2009; Prota et al., 2012), NCX3 (Heise et al., 2011), and UT-A1 (Naruse et al., 1997; Li et al., 2008), are regulated by glucocorticoids. In kidney, glucocorticoid receptor expression is found along the entire nephron including collecting duct epithelial cells (Ackermann et al., 2010), where the AQP2 is located. However, the influence of glucocorticoid hormones on AQP2 regulation is undetermined. Different groups report different results; some are even contradictory (Saito et al., 2000, 2009; Klein et al., 2006; Li et al., 2008). Previous investigations of dexamethasone on AQP2 are largely from *in vivo* studies. Obviously multiple factors may directly or indirectly affect AQP protein expression and activity, which add to the difficulty of evaluating the role of dexamethasone on AQP2 regulation. Therefore, the direct effects of glucocorticoid (dexamethasone) on AQP2 could be shadowed or influenced by secondary physiologic changes that occur *in vivo*. For example, in an *in vivo* study, dexamethasone at dose of 100 μg/100 g body wt causes hyperglycemia and glucosuria, which could also impair urinary dilution and concentration (Li et al., 2008). Dexamethasone treatment increases urine flow rate, urinary sodium excretion, and urinary urea excretion (Li et al., 2008). Glucocorticoids activate the serum glucocorticoid kinase 1 (SGK1) gene, which regulates sodium transporters, leading to hypertension (Vallon and Lang, 2005). The global effect of dexamethasone might explain why different groups observe different, or even contradictory, results of AQP2 regulation by dexamethasone when using different animal models (Saito et al., 2000, 2009; Chen et al., 2005; Klein et al., 2006). To specifically focus on the direct effect of dexamethasone on AQP2, in the current study, we used rat IMCD suspensions and treated them with dexamethasone. Our study clarified an important dispute and showed that dexamethasone upregulates AQP2 protein expression. This conclusion was further confirmed in HEK293 cells transfected with an AQP2 cDNA.

AQP2 is the vasopressin-sensitive water channel and vasopressin enhances AQP2 protein abundance (Hasler et al., 2002; Nedvetsky et al., 2010). Consistently, our data in Figure 1A show that vasopressin, as well as the adenylyl cyclase stimulator forskolin, upregulate AQP2 protein expression. This prompted us to ask whether dexamethasone upregulates AQP2 through an increase of vasopressin and/or the PKA pathway. However, it is unlikely that dexamethasone increases AQP2 by vasopressin *in vivo*. Both animal and human studies have demonstrated that synthesis and release of arginine vasopressin are actually suppressed by exogenous administration of glucocorticoid hormone under normal conditions and are increased in glucocorticoid deficiency (Raff, 1987; Biewenga et al., 1991; Toftegaard and Knudsen, 1995; Batalhão et al., 2008; Saito et al., 2009; Coiro et al., 2011). This suggests that glucocorticoids may have a direct effect on AQP2 and increase its expression.

Glucocorticoids are the hormones that generally promote protein catalysis and protein ubiquitination (Debigaré and Price, 2003; Sun et al., 2008). Despite the downregulation effect of dexamethasone on protein abundance, studies also show that dexamethasone can increase the expression of some proteins (Martinho et al., 2012; Laste et al., 2013; Nostramo et al., 2013). Our group has worked on kidney urea transporter UT-A1 for many years. We find that dexamethasone treatment decreases UT-A1 expression (Klein et al., 1997; Naruse et al., 1997). UT-A1 shares many of the same regulatory mechanisms as AQP2. Both UT-A1 and AQP2 are expressed in the kidney IM and are vasopressin sensitive targets. However, the protein abundance of UT-A1 and AQP2 sometimes change differently (Bou Matar et al., 2012). In fact, our initial desire was to investigate the mechanism of how dexamethasone downregulates UT-A1. Interestingly, opposite to the effect of dexamethasone on UT-A1, we found that dexamethasone treatment markedly increases AQP2 protein abundance in kidney IMCD (Figure 1) as well as in HEK293 cells transfected with AQP (Figure 2). This is consistent with Kortenoeven's finding that dexamethasone treatment increases AQP2 protein abundance in mouse collecting duct (mpkCCD) cells transfected with AQP2 (Kortenoeven et al., 2012). We further confirmed that the effect of dexamethasone on AQP2 protein expression is mainly dependent on the glucocorticoid receptor (Figure 3).

Protein degradation is an important mechanism by which cells regulate the levels of cellular proteins (Ciechanover, 2005). Both lysosomal and proteasomal degradation are involved in AQP2 protein degradation (Hasler et al., 2002; Nedvetsky et al., 2010; Kortenoeven et al., 2012). Treatment with a PKC stimulator induces AQP2 ubiquitination and lysosomal degradation (Tamma et al., 2011; Moeller et al., 2014). Inhibition of the lysosome by chloroquine inhibits AQP2 degradation (Kamsteeg et al., 2006; van Balkom et al., 2009; Kortenoeven et al., 2012). A study by Nedvetsky et al. showed that AQP2 also undergoes polyubiquitination and proteasome mediated protein degradation (Nedvetsky et al., 2010). Vasopressin activates cAMP-PKA and inhibits p38-mitogen-activated protein kinase (p38-MAPK). Inhibition of p38-MAPK is associated with decreased AQP2 polyubiquitination and proteasomal degradation, therefore leading to increasing AQP2 protein abundance (Nedvetsky et al., 2010). In the current study, we found that dexamethasone reduces the degradation of AQP2. Thus, we propose that dexamethasone increases the level of AQP2, at least partially, by reducing the degradation of AQP2. Kortenoeven et al. recently reported that prostaglandins reduce the dDAVP-induced AQP2 abundance and this effect can be prevented by lysosome inhibitor. Dexamethasone downregulates COX2 expression and prostaglandin synthesis (Kortenoeven et al., 2012). At least one mechanism of dexamethasone-upregulated AQP2 protein levels could be through inhibition of prostaglandin-induced AQP2 lysosomal degradation. Further study is required to elucidate whether inhibition of a proteasomal pathway is also involved and whether it is mediated by protein ubiquitination.

Another interesting finding in this study is that dexamethasone increases AQP2 distribution in lipid raft microdomains on the cell membrane. The physiological significance of this change is currently not known. Lipid rafts have been implicated in the regulation of membrane proteins in many aspects, such as protein trafficking, stability and bioactivity (Simons and Ikonen, 1997; Lingwood et al., 2009; Chen et al., 2011). It is not clear from this study whether AQP2 moving into lipid rafts will increase AQP2 protein stability and lead to increased protein membrane abundance or the increased AQP2 accumulation in lipid rafts is associated with enhanced AQP2 function. Future studies will be required to address these interesting questions.

In summary, glucocorticoid hormones are important hormones that regulate body water homeostasis. Although several groups have engaged in pursuing this question, whether this hormone could influence water channel AQP2 is inconclusive. Our *ex vivo* study using kidney IMCD suspensions, as well as heterogeneously expressed AQP2 in HEK293 cells and oocyte expression system, showed that dexamethasone directly upregulates AQP2 protein expression. Dexamethasone promotes AQP2 protein expression mainly by inhibiting AQP2 degradation. We conclude that glucocorticoid hormones may participate in the regulation of body water balance by increasing AQP2 expression.

### Conflict of interest statement

The authors declare that the research was conducted in the absence of any commercial or financial relationships that could be construed as a potential conflict of interest.
